# Sharply Reduced but Still Heavy Self-Harm Burdens in Hubei Province, China, 1990–2015

**DOI:** 10.3390/ijerph15020391

**Published:** 2018-02-24

**Authors:** Jingju Pan, Lan Zhang, Yumeng Tang, Qian Li, Chuanhua Yu, Tianjing He

**Affiliations:** 1 Institute of Chronic Non-Communicable Disease Control and Prevention, Hubei Provincial Center for Disease Control and Prevention, Wuhan 430079, China; jpan@hbcdc.com (J.P.); lan_zhang@hbcdc.com (L.Z.); ym_tang@hbcdc.com (Y.T.); qian_li@hbcdc.com (Q.L.); 2 School of Health Sciences, Wuhan University, Wuhan 430071, China; yuchua@whu.edu.cn

**Keywords:** self-harm burden, mortality, years of life lost (YLLs), prevalence, years lived with disability (YLDs), disability-adjusted life-years (DALYs), suicide method

## Abstract

The aims of this study were to describe fatal and non-fatal self-harm burdens, as well as burdens from the main preventable risk factors, and to investigate the different suicide methods in Hubei province in central China utilizing data from both Global Burden of Disease Study 2015 and Hubei Disease Surveillance Points system. All self-harm burdens including mortality, years of life lost (YLLs), prevalence, years lived with disability (YLDs), and disability adjusted life-years (DALYs) consistently demonstrated downward trends in Hubei from 1990 to 2015, with a bigger decline gap observed among females and narrower decreasing amplitudes among the elderly. Hubei experienced much higher age-standardized rates for self-harm mortality (22.0 per 100,000), YLLs (560.1 per 100,000) and DALYs (563.9 per 100,000) than the national (9.0, 292.3 and 295.0 per 100,000 respectively) and global levels (11.5, 453.3 and 457.9 per 100,000 respectively) in 2015. Self-harm burdens have begun shifting from females to males and the elderly suffered more self-harm burdens than other age groups. Alcohol use accounted for 20.9% of all self-harm DALYs for males, whereas intimate partner violence accounted for 24.4% of all self-harm DALYs for females. Poisoning, mainly pesticide self-poisoning, was still the most common method of suicide. Effective interventions by multi-sectoral collaboration are urgently needed to reduce the alarmingly heavy self-harm burdens in Hubei.

## 1. Introduction

Self-harm is a prevalent and complex problem worldwide, involving many psychological, social, cultural aspects, etc. Broadly speaking, self-harm is defined as an act of intentionally causing harm to oneself, irrespective of the type, motive or suicidal intent [1,2]. The outcomes of self-harm include non-fatal and fatal self-harm/suicide. Globally, it was estimated that 1.7 million outpatients, 1.5 million inpatients, and 842,000 deaths were attributable to self-harm in 2013 [3]. Self-harm was the second leading cause of death from injury and a main contributor to disability-adjusted life-years (DALYs) lost by injury globally [3,4]. Enormous burdens incurred by self-harm have been placed on health systems, especially in low- and middle-income countries, which accounted for 75% of the global suicide burden. Globally, half of all self-harm deaths happened in China and India [5].

In China, in 2015 alone, there were an estimated 796,000 cases of self-harm, leading to 135,000 deaths, ranking the tenth biggest cause of years of life lost (YLLs), resulting in as many as 4,578,000 DALYs [4,6]. Nonetheless, China is a vast and populous country with considerably heterogenous geographical distribution, economic development, and epidemiological transition. Briefly, China has been divided into the following six regions: North, East, Central, South, Northwest and Northeast, based on Chinese National Bureau of Statistics categorizations [7]. Spatiotemporal trend analysis pertaining to suicide showed the obvious disparity of suicide among these different regions in China. The highest self-harm death rates were seen in central China [7]. 

Hubei province is located in central China, with a land area of 185,900 square kilometers, and 58.5 million residents in 2015 [8,9]. According to Zhou’s research, China can be divided into five distinct groups based on the epidemiological characteristics [10]. Hubei province was categorized into the second group with relatively high life expectancy. However, this research also revealed that Hubei province had the highest self-harm death rate among all provinces in China [10]. Data from the Disease Surveillance Points (DSPs) system in Hubei showed that self-harm was the leading cause of death from injuries in rural areas from 2006 to 2008 [11,12]. Despite the high mortality rate shown, the whole picture of self-harm/suicide burdens in Hubei remains relatively unclear due to the lack of data. In order to support evidence-based priority setting for self-harm prevention and control at the provincial level, it is necessary and urgent to thoroughly assess its burdens in Hubei province.

Therefore, our first aim was to describe fatal and non-fatal self-harm burdens, as well as burdens from main preventable risk factors due to self-harm. The second aim was to describe the different suicide methods among people in Hubei. Findings from this study will add to the relatively limited information currently available about self-harm burdens among the population in Hubei, as well as identifying people at high risk and the most used suicide method in order to reduce self-harm burdens.

## 2. Materials and Methods

Data from two sources were used for this study. Sub-national data of Hubei province from Global Burden of Disease Study (GBD) 2015 were extracted and analyzed for self-harm burdens. GBD 2015 is the most comprehensive observational epidemiological study, utilizing diverse data sources and statistical models to calculate health estimates and making data comparable over time and across populations [4,6,13]. The main data sources included Disease Surveillance Points, Maternal and Child Surveillance System, Chinese Center for Disease Control and Prevention Cause of Death Reporting System [4,5]. Cause of Death Ensemble modelling (CODEm) was the principal method adapted to estimate mortality and YLLs; meanwhile, Bayesian meta-regression analysis using the DisMod-MR 2.1 software was used for prevalence and years lived with disability (YLDs) [4,6]. DALYs were computed by the summation of YLLs and YLDs [13]. Comparative risk assessment framework approaches were used to evaluate burdens of risk factors [14]. Fatal self-harm (numbers of deaths, YLLs), non-fatal self-harm (prevalence, YLDs) as well as DALYs (a summary measure of fatal and non-fatal disease outcomes) caused by self-harm were computed for the years 1990, 1995, 2000, 2005, 2010, and 2015 in Hubei province. Period analysis during 1990–2015 was carried out to estimate the age and sex specific changes of self-harm burdens. We compared self-harm burdens (age-standardized rates for mortality, YLLs, prevalence, YLDs, and DALYs using the GBD 2015 global standard population) in Hubei province to national and global levels [4]. Comparisons among them provide insights into the severe situation of self-harm burdens in Hubei. Age and sex specific percentages of total DALYs from risk factors attributed to self-harm were also calculated.

Data from Hubei DSPs in 2015 were used to analyze suicide method differences. The Hubei DSPs system is a sample-based mortality surveillance system, yielding representative provincial mortality [15]. In 2015, the Hubei DSPs system covered 13 million people, accounting for more than 20% of the whole population. Self-harm deaths/suicides were coded using the International Classification of Diseases 10th revision (ICD-10) (coding from X60 to X84 and Y87.0). Suicide methods were classified into seven categories: poisoning (X60–X69), hanging (X70), drowning (X71), firearms and explosive material (X72–X75), cutting or piercing instruments (X78), jumping from a high place (X80), other or unknown method (X76, X77, X79, X81–X84, X87.0). In short, cutting or piercing instruments were relabeled as cutting; jumping from a high place as jumping; other or unknown method as other methods in the text. In our study, no one utilized firearms and explosive material as a suicide method. The population from the sampled areas was used as a denominator while calculating the mortality rate. The χ^2^ test was performed to compare the method differences used by suicides between sexes and urban/rural areas. All statistical analyses were performed using SAS V9.4 (SAS Institute, Cary, NC, USA). The statistical significance level was defined as *p* < 0.05.

## 3. Results

### 3.1. Fatal Outcomes Attributable to Self-Harm

Table 1 presents the distributions of all and sex-specific fatal outcomes caused by self-harm from 1990 to 2015, including deaths, mortality rate, YLLs, and YLLs rate. The number of self-harm deaths dropped from 22,685 in 1990 to 13 173 in 2015, while the mortality rate decreased from 41.9 per 100,000 in 1990 to 23.9 per 100,000 in 2015. The number of self-harm deaths and mortality rate dropped by 41.9% and 43.1%, respectively from 1900 to 2015. Females experienced greater reduction for self-harm deaths (53.7%) and mortality rate (54.6%) than males (27.9% and 29.5%). Similarly, the YLLs and YLLs rate caused by self-harm from 1990 to 2015 in Hubei province also demonstrated decreasing trends. The YLLs and YLLs rate dropped by 64.5% and 65.2% respectively from 1990 to 2015, with 54.5%, 56.0% for males, and 72.2%, 72.8% for females (Table 1).

**Figure 1:**
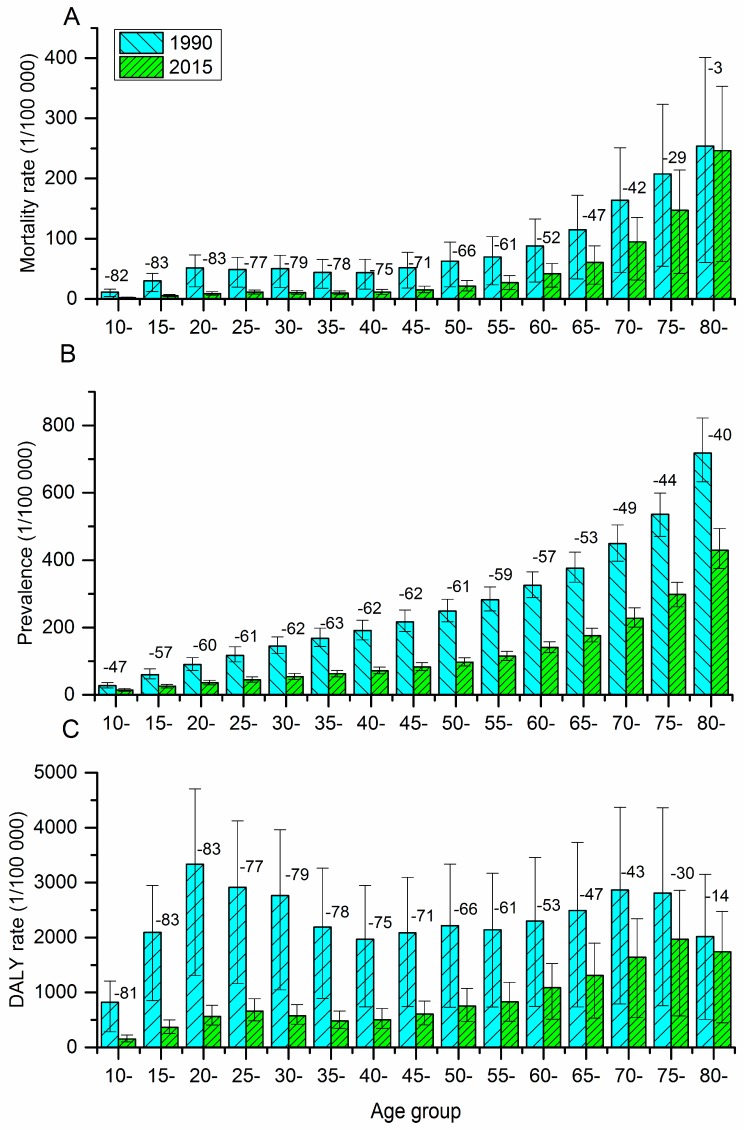
Mortality rates (**A**), prevalence (**B**), and DALYs rates (**C**) of self-harm in 1990 and 2015 by 5-year age intervals. Error bars indicate 95% uncertainty intervals. (Numbers shown above bars are percentage change for each age group between 1990 and 2015. DALY = disability-adjusted life-years.)

Changes in self-harm mortality rates varied widely by age in Hubei from 1990 to 2015 (Figure 1A). Self-harm mortality rates among people under 50 years old declined by more than 70%. However, the decreased amplitude was narrowed with aging. The least decreased amplitude was demonstrated in people above 80 years—dropped by only 3%—while the most decreased amplitude was observed in people between 15 and 24 years old—dropped by 83%. The self-harm mortality rates in 2015 remained high for people above 65 years old, peaking at 246.3 per 100,000 for people over 80 years old.

**Figure 2:**
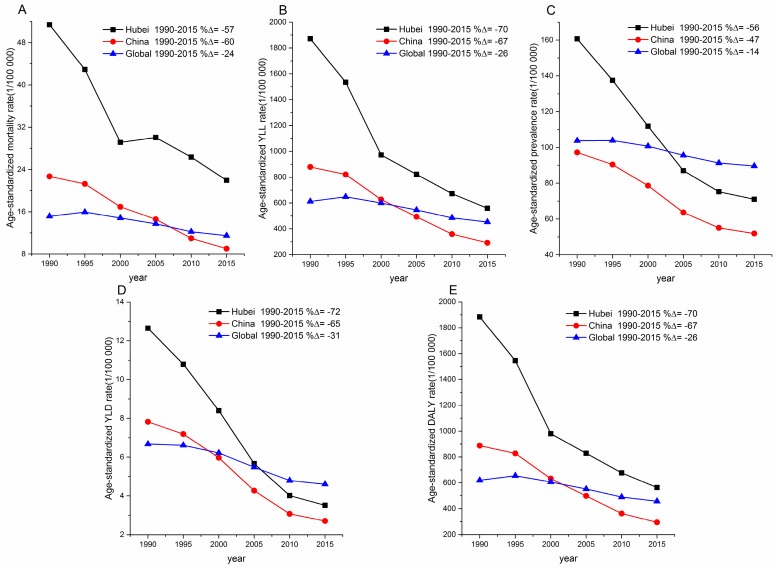
Age-standardized rates for mortality (**A**), years of life lost (YLLs) (**B**), prevalence (**C**), years lived with disability (YLDs) (**D**), and disability-adjusted life-years (DALYs) (**E**) per 100,000 due to self-harm in Hubei, China and globally, 1990–2015. %△ = percentage change.

Figure 2A,B shows the age-standardized rates (ASRs) for self-harm mortality and YLLs over time in Hubei, China and globally. The decreasing amplitudes of ASRs for mortality (57%) and YLLs (70%) in Hubei were similar to the national level (decreased by 60% and 67% for mortality and YLLs, respectively), but much larger than the global level (24% and 26% for mortality and YLLs, respectively). Despite the large decreasing amplitude above mentioned, Hubei still had much higher ASRs for mortality (22.0 per 100,000) and YLLs (560.1 per 100,000) than the national (9.0 and 292.3 per 100,000 for mortality and YLLs, respectively) and the global level (11.5 and 453.3 per 100,000 for mortality and YLLs, respectively) in 2015.

### 3.2. Non-Fatal Outcomes Attributable to Self-Harm

Table 2 provides an overall comparative view of all age patients, prevalence rate, YLDs and YLDs rate attributable to self-harm from 1990 to 2015. The number of patients from self-harm dropped from 68,368 (1990) to 45,408 (2015), with the prevalence rate decreasing from 126.4 per 100,000 (1990) to 82.3 per 100,000 (2015). Both number of patients and prevalence rate dropped by 33.6% and 34.9%, respectively from 1990 to 2015. The number of patients and prevalence rate of self-harm for males saw a 25.9% and 27.5% decrease from 1990 to 2015 respectively, while for females this decrease was 41.3% and 42.4% respectively. From 1990 to 2015, both YLDs and YLDs rate caused by self-harm in Hubei consistently decreased. Overall, YLDs and YLDs rate dropped by 61.2% and 62.0% respectively from 1990 to 2015: 56.6% and 57.5% for males, 65.0% and 65.7% for females (Table 2).

Between 1990 and 2015, prevalence rates attributed to self-harm among different age groups all decreased (Figure 1B). The decreased amplitudes among different age groups were all above 40%. The prevalence rates were increased with aging in Hubei in 2015. People above 80 years had the highest prevalence rate (429.0 per 100,000) for self-harm in 2015, while the lowest prevalence rate was observed among children less than 15 years old (14.6 per 100,000).

Figure 2C,D shows the ASRs for self-harm prevalence and YLDs over time in Hubei, China and globally. Much bigger decreasing amplitudes of ASRs for prevalence (−56%) and YLDs (−72%) were observed in Hubei than national (−47% and −65% for prevalence and YLDs, respectively) and global levels (−14% and −31% for prevalence and YLDs, respectively). Although ASRs for prevalence (71.0 per 100,000) and YLDs (3.5 per 100,000) caused by self-harm in Hubei, in 2015, were lower than the global level (89.7 and 4.6 per 100,000 for prevalence and YLDs, respectively), they were still higher than the national level (51.9 and 2.7 per 100,000 for prevalence and YLDs, respectively) in China.

### 3.3. DALYs Attributable to Self-Harm

**Figure 3:**
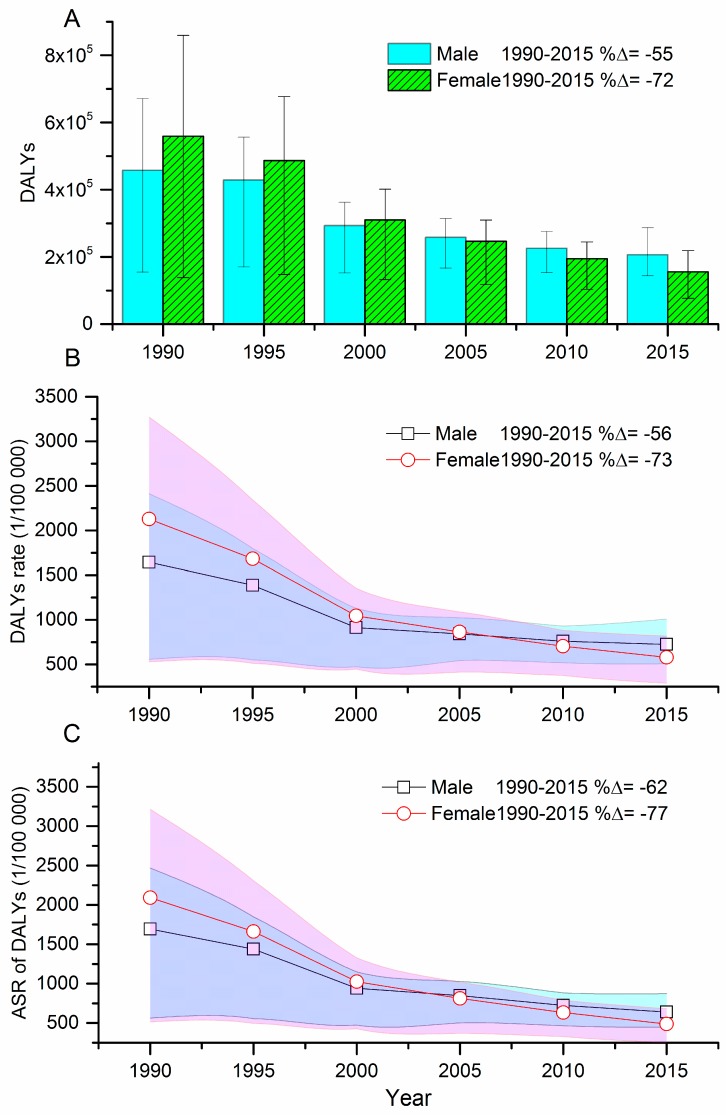
Disability adjusted life-years (DALYs) (**A**), DALYs rate (**B**) and age-standardized DALYs rate (**C**) per 100,000 due to self-harm in Hubei, 1990–2015. (%△ = percentage change. DALY = disability-adjusted life-years.)

Figure 3 shows the gender-specific trends of DALYs, DALYs rates and ASRs for DALYs by self-harm from 1990 to 2015 in Hubei. Both males and females demonstrated downward trends for DALYs and the corresponding rates. The DALYs rate for males fell by 56% from 1644.7 per 100,000 in 1990 to 724.3 per 100,000 in 2015, while there was a 73% drop for females from 2128.4 per 100,000 in 1990 to 580.5 per 100,000 in 2015. Similar reduction patterns were also illustrated in ASRs of DALYs for males and females (Figure 3C). From 2005, both DALYs and the age-standardized DALYs rate for males surpassed those of females.

Compared with 1990, DALYs rates caused by self-harm among various age groups in 2015 were all decreased (Figure 1C). Consistent with mortality rate, the decreased amplitude of the self-harm DALYs rate between 1990 and 2015 was also narrowed with aging. The least decreased amplitude was demonstrated in people above 80 years—dropped by only 14%—while the most decreased amplitude was observed in people between 15 and 24 years old—dropped by 83%. People above 60 years old in 2015 had a higher DALYs rate than their younger groups.

The age-standardized DALYs rate in Hubei, 2015 (563.9 per 100,000) was higher compared with national (295.0 per 100,000) and global levels (457.9 per 100,000) (Figure 2E). 

### 3.4. Risk Factors Attributable to Self-Harm

Table 3 shows the percentage of DALYs attributable to risk factors of self-harm by year, gender, and age group in Hubei. The proportion of self-harm DALYs attributable to alcohol and drug use, sexual abuse and violence, and their overlaps accounted for 30.9% for all ages in 2015. The percentage of DALYs from alcohol and drug use, sexual abuse and violence due to self-harm was different from 1990 to 2015. While the proportion of childhood sexual abuse slightly declined, and the proportion of drug use and intimate partner violence (IPV) remained stable, the proportion of alcohol use increased steadily.

The proportion of self-harm DALYs attributable to alcohol and drug use, sexual abuse and violence was lower among adolescents and the elderly. The proportion of alcohol use remained high among the middle age group; the proportion of drug use peaked among people aged 20–24 years; the proportion of childhood sexual abuse and IPV peaked for people aged 25–34 years and 35–44 years respectively. 

The proportion of self-harm DALYs attributable to alcohol and drug use, sexual abuse and violence was totally different among males and females in Hubei. Alcohol use played a bigger role (20.9%) as the cause of DALYs, and sexual abuse and violence accounted for a lower percentage (8.8%) for males in Hubei; for females, alcohol and drug use accounted for 3.9%, childhood sexual abuse 5.7%, and IPV 24.4%. 

### 3.5. Variation in Suicide Methods

Poisoning was the most common method of suicide, accounting for 63.1% (14.1 per 100,000 for mortality rate) of all suicides for all ages. Poisoning was predominated by pesticide self-poisoning, responsible for 60% of all self-harm deaths, with less than 3% from other poisoning. Then suicide methods were followed by hanging (25.2%, 5.6 per 100,000), drowning (5.2%, 1.2 per 100,000), jumping (4.9%, 1.1 per 100,000), cutting (0.9%, 0.2 per 100,000) and other methods (0.6%, 0.1 per 100,000) (Table 4). The four methods, i.e., poisoning, hanging, drowning and jumping, used by people who have self-harmed accounted for 98.4% of all methods. 

Males had higher mortality rates for hanging (6.2 vs. 5.0 per 100,000), cutting (0.3 vs. 0.1 per 100,000) and jumping (1.4 vs. 0.8 per 100,000) than females (*p* < 0.05). This pattern was observed in urban areas, but not rural areas, which did not show any difference between males and females in suicide methods.

Residents in rural areas had a higher suicide rate than their urban counterparts (25.6 vs. 19.9 per 100,000, *p* < 0.05) in Hubei, 2015. Rural residents had higher rates in poisoning (17.0 vs. 12.0 per 100,000, *p* < 0.05) and drowning (1.4 vs. 1.0 per 100,000, *p* < 0.05), but lower rates in jumping (0.8 vs. 1.3 per 100,000, *p* < 0.05) than urban residents. Urban men had a higher jumping rate than rural men (1.7 vs. 1.0 per 100,000, *p* < 0.05), while urban women had a lower drowning rate than rural women (0.8 vs. 1.7 per 100,000, *p* < 0.05).

## 4. Discussion

Sharply reduced but still alarmingly heavy self-harm burdens in Hubei, China, are illustrated for the first time in the present paper. All self-harm burdens including mortality, prevalence and DALYs in Hubei demonstrated obvious downward trends from 1990 to 2015. 

Even though obvious downward trends were demonstrated, self-harm burdens remain a key issue in Hubei. Our results demonstrated that Hubei bore higher self-harm burdens than national and global levels. The self-harm mortality rate in Hubei was twice that of the whole country in China and the world, and also much higher than other countries, e.g., USA and Australia [16,17]. A national study in China further revealed substantial geographic variation in suicide rates. Central China, including Hubei, had the highest suicide rates compared with other regions, with socioeconomic characteristics, and psychological and mental factors probably underlying this variation [7]. The reasons causing such heavy self-harm burdens in Hubei are not yet clear. Biological, psychological, social, environmental and cultural factors have been proved important in determining suicidal behaviors [18]. A few qualitative research studies pertaining to suicide in Hubei suggested that changes in family structure, inter-generational relations, as well as people’s attitude towards life might be the key elements for suicidal behavior [19,20,21]. The patterns and characteristics in family structure in Hubei have changed from the traditionally big family towards a small, centralized one, resulting in the traditional value of fostering the elderly by the family possibly being substantially weakened [21]. In addition, there was a cultural phenomenon normalizing suicide in some deprived rural areas of Hubei where people treated death with arbitrary attitudes, lacked reverence towards life and widely held the conception that people “should die when they are useless” [20]. Those factors together provided a forgiving, even encouraging, social and psychological milieu for people to end their lives by self-harming, especially the elderly [20]. Interpersonal conflict, suffering from serious diseases and economic hardships were identified as the three leading causes for completed suicide among rural females in Hubei by some researchers [22].

Our study showed that both men and the elderly were the two main groups that suffered more self-harm burdens than others. Females in Hubei used to be in disadvantaged positions and had higher self-harm burdens than males, similar to the national situation in China [7,18]. Nonetheless, our results revealed that females experienced a bigger reduction in self-harm burdens than males from 1990 to 2015 in Hubei, consistent with the national results in China [3,23,24]. Since 2010, self-harm burdens have begun to shift from females to males. Males surpassed females in self-harm burdens. Some researches attributed the big declining trends among females to the process of urbanization and economic development during the past decades [25,26]. Meanwhile, in the current social vicissitudes of China, fierce work stress, and anxiety due to a rapid rise in living cost may put more pressure on males; these factors are not conducive to their mental health and make them susceptible to mental illness, a key risk factor for suicide.

Self-harm burdens among different age groups all dropped consistently from 1990 to 2015. However, the least decreased amplitudes were demonstrated among the elderly. Self-harm burdens increased with aging, and remained at higher levels among the elderly in Hubei, 2015, which is consistent with most parts of the world [18]. As the society is ageing rapidly, the self-harm burdens among the elderly in Hubei may be even worse. By the end of 2016, the latest data from Bureau of Statistics in Hubei show that the proportion of the population aged 65 years and over accounted for 10.8% of the total population. Besides, deaths resulting from suicide are sometimes misreported as accidents or natural causes, for all kinds of reasons, such as traditional and cultural backgrounds which see suicide as a stigma, or insurance policies in which medical or other expenses are not covered or reimbursed by insurance companies for people dying of suicide [18,27]. What is more, the probability of suicide death being classed as other diseases among the elderly might be slightly higher than other age groups due to age effects. These factors may put the elderly people in Hubei at high risk for suicide. As a result, the continued severity of self-harm burdens among the elderly in Hubei might be expected.

By analyzing risk factors pertaining to self-harm, alcohol use and IPV were identified as the main risk factors attributing to self-harm burdens for males and females respectively. This obvious gender difference in percentage of risk factors accounting for total DALYs could be explained by high levels of alcohol consumption in men compared with women. The rate of drinkers in Hubei was nearly five times higher for men (48.8%) than women (10.6%) [28]. Because of Chinese cultural norms, social status and other factors, it is accepted for men to drink more than women [29]. Research has consistently shown a strong association between self-harm behavior and alcohol misuse [30]. IPV was identified as the most robust risk factor for self-harm among women in Hubei. Although both men and women may be victims of IPV, it is more common for men to perpetrate violence against women [31]. IPV is the most common form of violence against women, with global lifetime prevalence rates ranging from 15% to 71% [32]. In China, a prevalence of 51.6% physical and/or sexual IPV was reported in the surveyed sites [33]. Higher rates of self-harm behaviors and self-harm ideation have been observed in female victims of IPV compared with nonvictims [34]. In rural China, one-third of women visiting emergency rooms due to serious self-harm were disclosed to be victims of IPV [35]. The reasons for victims of IPV turning to self-harm in China might be to air painful emotions caused by abuse or as a last resort to escape by dying when they see no other options and are no longer able to endure the unbearable pain and suffering [36]. In order to prevent self-harm triggered by IPV, social support has been identified as a key protective factor [37]. Clinicians that encounter victims of IPV should make an effort to mobilize social support and facilitate referrals to legal, medical, and psychosocial services [37].

By investigating method-specific suicide in Hubei, our study found that poisoning, mainly pesticide suicides which accounted for approximately 60% of all suicides, was the most common suicide method. The proportion of fatal pesticide self-poisoning in Hubei was considerably higher than national (49%) and global levels (13%) and other regions worldwide (e.g., high income countries (1.7%), low and middle income countries in Europe (0.9%)) [38,39]. The pattern of suicide methods in Hubei was similar to the national pattern in China [38], but different from other countries, e.g., firearms being the main suicide method in the USA, hanging in Australia, jumping in Singapore [16,17,40,41]. Restricting access to highly lethal means has been proved to be effective in preventing suicide. Given the severity of fatal self-poisoning by pesticide, further strict management of lethal pesticides in Hubei still needs to be implemented in order to reduce the high burden of fatal self-harm. 

Our study has important public implications. In 2015, the Sustainable Development Goals (SDGs) were adopted by the UN General Assembly [42]. They cover 17 universal goals, 169 targets and 230 indicators. One of 230 indicators from the SDGs is to reduce the number of self-harm deaths by one-third by 2030 compared to 2015. Both Health China 2030 and Health Hubei 2030 implemented by central and provincial government also emphasize the prevention and control of self-harm death. To accomplish this, Hubei faces a big challenge. Faster and more inclusive progress is urgently needed. Many lives can be saved. Therefore, time is of the essence. Besides restricting access to means of suicide, basic mental health services, and social support networks might need to be strengthened in Hubei [43,44].

Our study has some limitations. Firstly, the general limitations outlined in the GBD 2015 analytical approach apply to the present study [4,6,13]. Secondly, there might be an underestimation of self-harm burdens in Hubei, because of underreporting and miscoding issues. Self-harm deaths might be reported as deaths of undetermined intent, accidents and ill-defined and unknown cause of mortality [18]. A national evaluation of the accuracy of reported suicides in the Chinese population showed that 5.4% of “other” external causes, 48.6% of “unknown” external causes and 15.0% of deaths attributed to psychiatric disorders should be re-classified as suicide [27]. Lastly, the suicide burden and its secular trends among the population in Hubei were analyzed at the whole provincial level. We did not compare the difference of self-harm burdens between various municipalities. Given the severity of self-harm burdens in Hubei, research with regard to self-harm burdens at the municipality and county level will be our next priority in order to tackle this urgent public issue with area-specific measures.

## 5. Conclusions

Although self-harm burdens in Hubei province demonstrated clearly decreasing trends, the burdens are still substantially heavier than the national and global levels. Pesticide self-poisoning remains the leading suicide method, accounting for 60% of suicides. Due to the complexity of self-harm, effective interventions with multi-sectoral collaboration are urgently needed to reduce self-harm/suicide, targeting people who are at high risk of self-harm, especially males and the elderly. Further strict management of and restricted access to lethal pesticides are warranted.

## Figures and Tables

**Table 1:** Fatal outcome of self-harm in Hubei, 1990–2015.

Gender/Year	All Age Deaths	Mortality Rate (1/100,000)	YLLs	YLLs Rate (1/100,000)
**Total**				
1990	22,685 (8080–30,951)	41.9 (14.9–57.2)	1,010,770 (380,864–1,392,060)	1869.0 (704.2–2574.0)
1995	20,961 (7780–26,422)	35.0 (13.0–44.2)	909,755 (358,468–1,144,165)	1520.9 (599.3–1912.8)
2000	14,803 (7144–17,672)	24.0 (11.6–28.6)	598,279 (309,844–712,316)	969.8 (502.2–1154.6)
2005	15,167 (7512–17,947)	25.6 (12.7–30.3)	501,187 (301,344–589,108)	847.2 (509.4–995.8)
2010	14,244 (7367–17,073)	24.9 (12.9–29.8)	417,424 (274,119–493,941)	728.5 (478.4–862.0)
2015	13,173 (6786–16,749)	23.9 (12.3–30.3)	359,034 (236,934–454,291)	650.5 (429.3–823.1)
Percent change between 1990 and 2015	−41.9%	−43.1%	−64.5%	−65.2%
**Males**				
1990	10,364 (3357–15,127)	37.3 (12.1–54.4)	454,880 (152,382–668,153)	1635.5 (547.9–2402.2)
1995	9910 (3752–12,795)	32.0 (12.1–41.4)	426,374 (166,614–553,241)	1378.8 (538.8–1789.1)
2000	7201 (3524–8836)	22.5 (11.0–27.6)	290,706 (149,907–359,895)	906.9 (467.6–1122.7)
2005	7715 (4136–9436)	25.2 (13.5–30.8)	256,382 (164,900–311,778)	836.1 (537.8–1016.7)
2010	7597 (4096–9384)	25.7 (13.8–31.7)	223,879 (151,957–274,098)	756.0 (513.1–925.5)
2015	7470 (4237–10,112)	26.3 (14.9–35.6)	204,758 (142,795–285,078)	720.3 (502.3–1002.9)
Percent change between 1990 and 2015	−27.9%	−29.5%	−54.5%	−56.0%
**Females**				
1990	12,322 (2901–19,042)	46.9 (11.0–72.5)	555,890 (135,168–855,785)	2116.2 (514.6–3257.9)
1995	11,051 (3115–15,213)	38.2 (10.8–52.7)	483,381 (144,602–674,562)	1673.1 (500.5–2334.8)
2000	7603 (2918–9909)	25.7 (9.8–33.4)	307,573 (130,235–399,707)	1037.8 (439.4–1348.6)
2005	7452 (2820–9507)	26.2 (9.9–33.4)	244,805 (116,072–308,111)	859.1 (407.3–1081.2)
2010	6647 (2654–8540)	24.0 (9.6–30.9)	193,546 (102,402–243,165)	699.1 (369.9–878.4)
2015	5703 (2173–8163)	21.3 (8.1–30.5)	154,277 (76,143–217,569)	576.4 (284.5–812.8)
Percent change between 1990 and 2015	−53.7%	−54.6%	−72.2%	−72.8%

YLLs = years of life lost.

**Table 2:** Non-Fatal outcome of self-harm in Hubei, 1990–2015.

Gender/Year	Number of Patients	Prevalence Rate (1/100,000)	YLDs	YLDs Rate (1/100,000)
**Total**				
1990	68,368 (60,673–78,307)	126.4 (112.2–144.8)	5772 (4102–7784)	10.7 (7.6–14.4)
1995	66,279 (59,058–75,242)	110.8 (98.7–125.8)	5552 (3916–7460)	9.3 (6.5–12.5)
2000	58,856 (52,534–66,327)	95.4 (85.2–107.5)	4702 (3309–6350)	7.6 (5.4–10.3)
2005	49,236 (44,305–54,982)	83.2 (74.9–92.9)	3367 (2372–4535)	5.7 (4.0–7.7)
2010	45,314 (41,082–50,304)	79.1 (71.7–87.8)	2479 (1718–3370)	4.3 (3.0–5.9)
2015	45,408 (41,079–50,364)	82.3 (74.4–91.3)	2237 (1525–3066)	4.1 (2.8–5.6)
Percent change between 1990 and 2015	−33.6%	−34.9%	−61.2%	−62.0%
**Males**				
1990	34,329 (30,688–38,641)	123.4 (110.3–138.9)	2580 (1818–3449)	9.3 (6.5–12.4)
1995	34,109 (30,594–38,172)	110.3 (98.9–123.4)	2552 (1795–3427)	8.3 (5.8–11.1)
2000	31,200 (28,160–34,698)	97.3 (87.8–108.2)	2238 (1587–3021)	7.0 (4.9–9.4)
2005	26,553 (24,123–29,419)	86.6 (78.7–95.9)	1622 (1138–2170)	5.3 (3.7–7.1)
2010	24,882 (22,711–27,352)	84.0 (76.7–92.4)	1212 (839–1647)	4.1 (2.8–5.6)
2015	25,436 (23,083–28,004)	89.5 (81.2–98.5)	1121 (773–1522)	3.9 (2.7–5.4)
Percent change between 1990 and 2015	−25.9%	−27.5%	−56.6%	−57.5%
**Females**				
1990	34,039 (29,714–39,714)	129.6 (113.1–151.2)	3191 (2235–4367)	12.1 (8.5–16.6)
1995	32,170 (28,315–37,157)	111.3 (98.0–128.6)	3000 (2110–4050)	10.4 (7.3–14.0)
2000	27,656 (24,465–31,684)	93.3 (82.5–106.9)	2464 (1710–3373)	8.3 (5.8–11.4)
2005	22,683 (20,230–25,787)	79.6 (71.0–90.5)	1745 (1210–2390)	6.1 (4.2–8.4)
2010	20,431 (18,306–23,041)	73.8 (66.1–83.2)	1267 (871–1730)	4.6 (3.1–6.2)
2015	19,973 (17,870–22,438)	74.6 (66.8–83.8)	1116 (749–1541)	4.2 (2.8–5.8)
Percent change between 1990 and 2015	−41.3%	−42.4%	−65.0%	−65.7%

YLDs = years lived with disability.

**Table 3:** Percentage of total DALYs from risk factors for self-harm by year, by 5-year age intervals and by gender in Hubei, 2015.

Risk Factors	Alcohol and Drug Use		Sexual Abuse and Violence	
	Alcohol Use, % (95%, UI)	Drug Use, % (95%, UI)	Childhood Sexual Abuse, % (95%, UI)	Intimate Partner Violence, % (95%, UI)
Years				
1990	7.7 (5.0–12.0)	2.8 (1.7–4.5)	9.2 (2.1–19.6)	9.9 (4.5–14.9)
1995	8.7 (6.3–12.9)	3.1 (1.9–4.9)	8.8 (2.0–18.7)	10.8 (5.1–15.5)
2000	8.9 (6.7–12.1)	3.0 (1.9–4.8)	8.4 (1.9–17.9)	10.5 (6.2–14.7)
2005	9.8 (7.5–13.1)	2.8 (1.8–4.3)	8.0 (1.8–17.2)	10.8 (6.6–14.9)
2010	11.3 (9.0–14.5)	2.9 (1.8–4.6)	7.5 (1.7–16.1)	10.9 (7.0–14.6)
2015	12.8 (9.8–16.6)	3.0 (1.9–4.7)	7.4 (1.6–16.0)	10.4 (6.3–14.8)
Age groups				
10-	0	0.1 (0.0–0.2)	6.3 (1.3–13.7)	0
15-	6.8 (3.8–11.2)	4.6 (2.2–8.7)	7.9 (1.7–17.1)	0.0 (0.0–0.1)
20-	8.9 (5.5–13.4)	7.6 (4.1–13.1)	8.9 (2.0–18.8)	3.5 (0.6–9.4)
25-	10.0 (6.2–14.6)	6.9 (4.0–11.4)	9.1 (2.1–19.1)	9.3 (1.8–22.3)
30-	11.3 (7.7–15.8)	5.1 (3.2–8.4)	9.1 (2.0–19.5)	12.9 (3.2–26.8)
35-	13.6 (8.9–19.5)	3.4 (2.2–5.4)	8.9 (1.9–18.7)	15.2 (3.5–31.9)
40-	14.9 (10.0–20.4)	2.4 (1.5–3.7)	8.6 (1.8–18.0)	15.2 (3.6–30.9)
45-	15.3 (10.2–21.0)	1.8 (1.1–2.8)	8.0 (1.7–17.1)	14.9 (3.6–29.9)
50-	15.2 (10.3–20.6)	1.6 (1.0–2.4)	7.5 (1.6–16.1)	13.9 (3.5–28.8)
55-	16.0 (10.7–22.1)	1.7 (1.1–2.6)	7.1 (1.5–15.5)	11.2 (2.6–24.2)
60-	14.0 (9.4–19.0)	1.9 (1.3–2.8)	6.7 (1.4–14.7)	9.5 (2.0–20.8)
65-	13.6 (9.4–18.9)	2.1 (1.3–3.1)	6.4 (1.4–13.9)	9.2 (2.0–20.7)
70-	13.2 (9.1–18.8)	2.1 (1.3–3.2)	6.0 (1.3–13.3)	8.3 (1.9–18.9)
75-	12.2 (8.2–17.7)	1.6 (1.0–2.5)	5.6 (1.2–12.5)	8.2 (1.5–19.5)
80-	7.6 (4.3–12.5)	0.4 (0.2–0.7)	4.1 (0.9–9.2)	9.4 (1.6–20.9)
Gender				
Male	20.9 (17.9–24.7)	3.7 (2.4–5.8)	8.8 (1.9–18.4)	0
Female	1.9 (1.5–2.3)	2.0 (1.3–3.1)	5.7 (1.2–12.6)	24.4 (17.8–31.2)
Both	12.8 (9.8–16.6)	3.0 (1.9–4.7)	7.4 (1.6–16.0)	10.4 (6.3–14.8)

Data in parentheses are 95% uncertainty intervals; DALYs = Disability-Adjusted Life-Years.

**Table 4:** External causes of fatal self-harm outcomes, percentage and mortality rates, by gender and area.

External Causes	Male		Female		Both Sexes	
	Percent (%)	Mortality Rate (1/100,000)	Percent (%)	Mortality Rate (1/100,000)	Percent (%)	Mortality Rate (1/100,000)
Totals						
Poisoning	59.1	13.5	67.5	14.7	63.1	14.1
Hanging	27.4	6.2 *	22.9	5.0	25.2	5.6
Drowning	5.1	1.2	5.4	1.2	5.2	1.2
Cutting	1.4	0.3 *	0.4	0.1	0.9	0.2
Jumping	6.2	1.4 *	3.6	0.8	4.9	1.1
Other methods	0.9	0.2	0.3	0.1	0.6	0.1
Totals	100.0	22.8	100.0	21.8	100.0	22.3
Urban						
Poisoning	54.9	11.3	66.1	12.7	60.2	12.0
Hanging	29.1	6.0 *	24.7	4.7	27.0	5.4
Drowning	5.4	1.1	4.3	0.8	4.8	1.0
Cutting	1.7	0.3 *	0.3	0.1	1.0	0.2
Jumping	8.5	1.7 *	4.4	0.8	6.5	1.3
Other methods	0.6	0.1	0.2	0.0	0.4	0.1
Totals	100.0	20.6	100.0	19.2	100.0	19.9
Rural						
Poisoning	63.7	16.5 **^#^**	68.9	17.5 **^#^**	66.2	17.0 **^#^**
Hanging	25.5	6.6	20.9	5.3	23.3	6.0
Drowning	4.8	1.2	6.5	1.7 **^#^**	5.6	1.4 **^#^**
Cutting	1.1	0.3	0.5	0.1	0.8	0.2
Jumping	3.7	1.0 **^#^**	2.7	0.7	3.2	0.8 **^#^**
Other methods	1.2	0.3	0.5	0.1	0.9	0.2
Totals	100.0	25.9 **^#^**	100.0	25.4 **^#^**	100.0	25.6 **^#^**

* *p* < 0.05 between different genders. **^#^** *p* < 0.05 between urban and rural areas.
